# Novel Iron-based ternary amorphous oxide semiconductor with very high transparency, electronic conductivity, and mobility

**DOI:** 10.1038/srep18157

**Published:** 2015-12-16

**Authors:** A. Malasi, H. Taz, A. Farah, M. Patel, B. Lawrie, R. Pooser, A. Baddorf, G. Duscher, R. Kalyanaraman

**Affiliations:** 1 Department of Chemical and Biomolecular Engineering, University of Tennessee, Knoxville, Tennessee, 37996, USA; 2 Bredesen Center, University of Tennessee, Knoxville, Tennessee 37996, USA; 3 Department of Material Science and Engineering, University of Tennessee, Knoxville, Tennessee 37996, USA; 4 Quantum Information Science Group, Computational Sciences and Engineering Division, Oak Ridge National Laboratory, Oak Ridge, TN 37831, USA; 5 Center for Nanophase Materials Sciences, Oak Ridge National Laboratory, Oak Ridge, TN 37831, USA; 6 Material Science and Technology Division, Oak Ridge National Laboratory, Oak Ridge, TN 37831, USA

## Abstract

Here we report that ternary metal oxides of type (Me)_2_O_3_ with the primary metal (Me) constituent being Fe (66 atomic (at.) %) along with the two Lanthanide elements Tb (10 at.%) and Dy (24 at.%) can show excellent semiconducting transport properties. Thin films prepared by pulsed laser deposition at room temperature followed by ambient oxidation showed very high electronic conductivity (>5 × 10^4^ S/m) and Hall mobility (>30 cm^2^/V-s). These films had an amorphous microstructure which was stable to at least 500 °C and large optical transparency with a direct band gap of 2.85 ± 0.14 eV. This material shows emergent semiconducting behavior with significantly higher conductivity and mobility than the constituent insulating oxides. Since these results demonstrate a new way to modify the behaviors of transition metal oxides made from unfilled d- and/or f-subshells, a new class of functional transparent conducting oxide materials could be envisioned.

Materials which combine large optical transparency with electronic conductivity are of great scientific interest, partly due to a dearth of such materials, and largely due to their potential for applications. For example, solar cells and smart windows rely upon having a transparent front conductor, while thin film transistors made from transparent semiconductors are central to flat panel displays, flexible optoelectronics devices, and organic light emitting devices^1^,^2^,^3^. In 2004 a new era in the design and application of these materials was ushered in when the Japanese team of Nomura *et al*. showed the room temperature fabrication of a high-performing thin film transistor made from Indium-based amorphous oxide semiconductor material^4^. This report led to widespread interest in amorphous conducting and semiconducting oxide materials because it demonstrated the capability of combining high optical transparency with high electronic conductivity and hall mobility (>10 cm^2^/V-s), which greatly exceeded the value for amorphous Si (<1 cm^2^/V-s)^2^. The amorphous microstructure makes such materials extremely attractive because they can be synthesized at room temperature thus reducing processing cost and complexity, can eliminate non-uniformity arising from defects such as grain boundaries seen in polycrystalline materials, and can show better tolerance to mechanical stress as compared to polycrystalline or crystalline materials^5^. Presently, all known high mobility (>10 cm^2^/V-s) amorphous oxide materials, such as In-Ga-Zn oxide (a-IGZO) and Zn-In-Sn oxide, are made by combining two or more oxides which have extremely high mobility arising from their metal cations having an oxidation state configuration given by (n-1)d^10^ns^0^, i.e. oxides with an s-conduction band. As postulated by Hosono *et al*. and verified by others, the large spatial extent and orientation independence of the spherically symmetric *ns* orbitals can result in extremely high mobilities and conductivity behavior^6^,^7^,^8^,^9^,^10^.

Here we show the first evidence of a ternary amorphous oxide semiconductor that goes beyond this existing paradigm of requiring metal cations with (n-1)d^10^ns^0^ in order to show good conduction. This novel oxide material consists of the metals Fe, Tb and Dy whose common oxidation states involve partly filled d-subshells (3d^**5**^4s^0^ for Fe) or f-subshells (4f^**9**^6s^**0**^ for Tb and 4f^**10**^6s^**0**^ for Dy) and are normally d- or f-band insulating oxides^11^,^12^. We found that films made at room temperature by pulsed laser deposition from a metallic target, followed by oxidation by ambient exposure, were amorphous, had very high visible light transparency (>90%), high thermal stability of the amorphous phase, very high electronic conductivity (>5 × 10^4^ S/m), and extraordinarily high as-prepared Hall mobilities of >30 cm^2^/V-s, a combination of properties that already rivals that of the best known Indium-based amorphous oxide semiconductors^2^.

## Results

**Figure 1:**
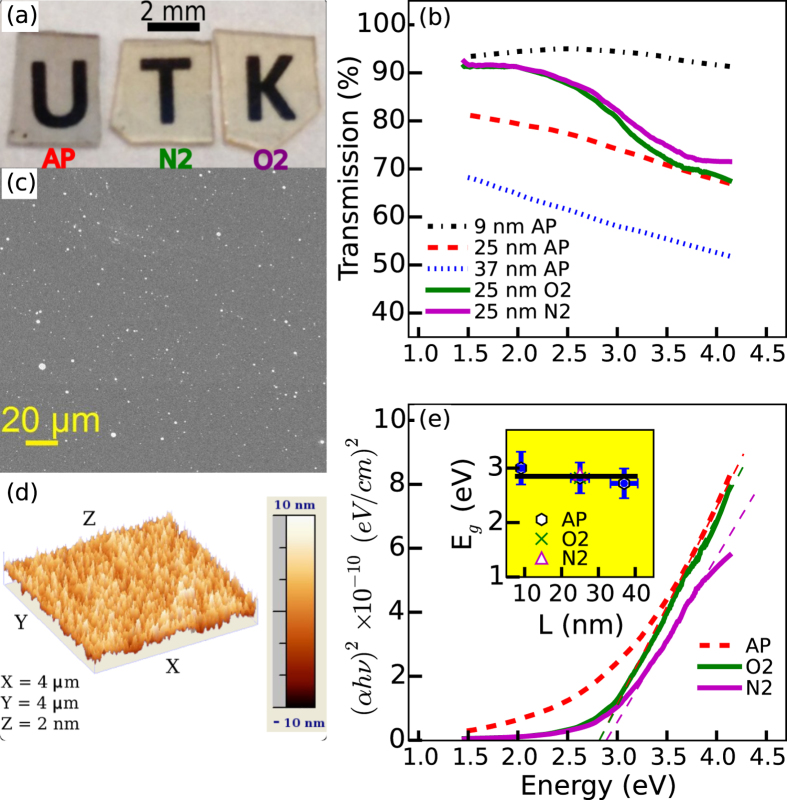
(**a**) Optical photograph of lettered blocks showing the large transparency of 25 nm thick films in as-prepared state (marked as AP), and following annealing in nitrogen (marked as N2) and oxygen (marked as O2) at 500 °C for 2 hrs. (**b**) Spectrally resolved transmission of as-prepared films with thickness between 9 to 37 nm (dashed curves) and following annealing of the 25 nm film (solid curves). (**c**,**d**) SEM (**c**) and AFM (**d**) information from a 25 nm as-prepared film. (**e**) Tauc plot comparing the direct optical absorption in 25 nm films (as prepared is dashed line while annealed are solid lines). The extrapolations from the strongly absorbing linear regimes are shown and were used to estimate the band gap. Inset shows the Tauc direct band gap values as a function of thickness L of the as-prepared films and following annealing of the 25 nm film. A line corresponding to the average band gap value from measurements of various as-prepared films is also shown in the inset.

Our investigations were motivated by the study of the magneto-optical properties of the giant magnetostrictive material terfenol-D [metal composition of Fe(65.7 at.%):Tb(10.3 at.%):Dy(24 at.%)] for applications in optical sensing and computing. Films of thickness from 9 to 37 nm were deposited by the pulsed laser deposition (PLD) technique onto quartz substrates under high vacuum conditions (~5 × 10^−8^ Torr base pressure) at room temperature. However, instead of a metallic sheen, these films showed optical transparency in their as-prepared (AP) state, as shown in Fig. 1(a) for the sample labeled AP. This transparency was quantitatively evaluated as a function of film thickness by transmission spectroscopy. The dashed curves in Fig. 1(b) show that the as-prepared films had a coefficient of transmission (T) of >50% in an energy range spanning the UV to NIR (1.5 to 4 eV) and could reach as high as T > 90% for the thinner films (9 nm thickness). We confirmed that this high transparency was not due to a discontinuous film morphology. Figure 1(c,d) show a scanning electron microscope image and an atomic force microscope image respectively of the 25 nm thick as-prepared film. The film was continuous with a random distribution of nanoparticles typical of the PLD process. The root mean square surface roughness of the film in the particulate free regions was estimated to be between 1 to 2 nm, depending on the film thickness [Fig. 3(c-inset)], as ascertained from the atomic force microscopy measurements. To further understand this optical transparency, we estimated the absorption coefficient (*α*) from *α* = −*ln*(*T* % /100)/*L*, where L is the film thickness and then generated the dependence of (*αhv*)^1/*m*^ versus *hv*, which represents the photon energy as the product of Planck’s constant (*h*) and photon frequency *v*, while *m* represents the type of absorption. This Tauc plot is shown in Fig. 1(e) for the case m = 1/2, i.e. for a direct allowed transition for the films of 25 nm thickness (as-prepared is dashed curve). The rapid change in the slope of the curve indicates large interband absorption and extrapolating this linear region permitted estimation of the direct allowed energy band gap 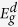. We found that the as-prepared film of L = 25 nm film had a 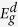 of 2.82 *eV*. Similar analysis of the other as-prepared films (see supplemental material) yielded values between 2.72 and 3.0 eV, as shown in the inset of Fig. 1(e), giving an average direct band gap of 2.85 ± 0.14 eV. No evidence for an indirect gap could be found from a similar Tauc plot analysis.

The optical data suggested that the films were very likely oxidizing rapidly upon exposure to air since the transparency was not consistent with forming metallic films (for comparison, films of metals like Au, Ag, Cu, Fe etc. achieve such high transmission at thicknesses of only a few nm). We further modified the oxidation state of the films by a high temperature anneal (500 °C for 2 hours) in either a N_2_-rich or O_2_-rich environment (air). The optical photograph in Fig. 1(a) shows that the transparency increased following annealing of 25 nm thick films (i.e. optical images marked as N_2_ and O_2_ corresponding to the N_2_ and O_2_ annealing). The qualitative increase was also evident from the optical transmission curves [Fig. 1(b), solid curves] for the annealed films in comparison to the as-prepared film. The Tauc plot analysis of the annealed samples [Fig. 1(e, solid lines)] yielded 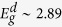 and 2.82 eV for the N_2_ and O_2_ samples respectively. These estimated band gap values were within the measurement uncertainty of the average value estimated for the as-prepared films, as seen in the inset of Fig. 1(e), implying that the high temperature anneal did not significantly influence the microstructure of the films. The optical behavior of the as-prepared and thermally annealed films pointed to an oxidized film that behaved like a semiconductor and one whose band gap was unchanged upon annealing to high temperatures.

**Figure 2:**
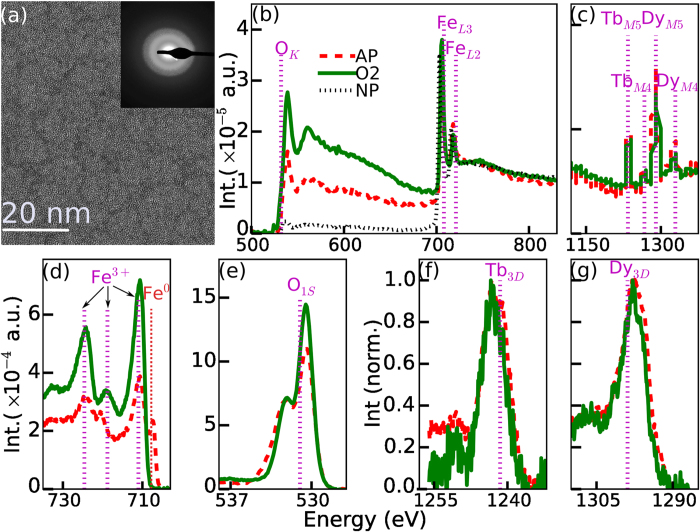
(**a**) Amorphous microstructure of the as-prepared films was evidenced by TEM imaging and selected area diffraction (inset). (**b**,**c**) EELS analysis of the as-prepared and O_2_ annealed films only detected Fe, and O (Fig. b) and Tb and Dy (Fig. c). In Fig. (b) the EELS spectrum from a PLD nanoparticle (NP) is also shown by dotted curve. (**d**–**g**) XPS measurements showing the various detected components in the as-prepared vs O_2_ annealed films. (**d**) Fe 2p signal (**e**) O 1s signal. (**f**) Tb 3d 5/2 signal, (**g**) Dy 3d 5/2 signal. In figures (**b–g**), the as-prepared (AP) films are shown by dashed curves while the O_2_ (O_2_) annealed films are shown by solid curves. The vertical dotted lines mark the position of the various absorption edges (EELS) and peaks (XPS) as indicated. The additional vertical lines in Fig. (d) correspond to the additional Fe_3d_ absorption peaks found in the hematite and magnetite form or iron oxides.

To understand the origin of this semiconducting behavior, we performed a detailed study of the structure and chemical composition of the as-prepared and O_2_ annealed films. Glancing incidence X-ray diffraction (GIXRD) from the target material used for the PLD process showed peaks corresponding to polycrystalline terfenol-D. However, the as-prepared films were featureless, indicating an amorphous microstructure, and remained so even following the 500 °C thermal treatments (see supplemental material). We next prepared films by PLD onto ultrathin membranes (C or Si_3_N_4_) for evaluation by transmission electron microscopy (TEM). Figure 2(a) shows that the typical microstructure of as-prepared films was amorphous, confirmed by the TEM diffraction pattern shown in the inset. A similar amorphous microstructure was evident for the 500 °C O_2_ annealed films (shown in supplemental material). Therefore, the TEM results along with the GIXRD observation independently established that the as-prepared and 500 °C O_2_ annealed films were amorphous. The chemical constituents, homogeneity, and composition of the films were measured by two different approaches: core-loss electron energy loss spectroscopy (EELS) in the TEM was used to obtain the film volume averaged information while X-ray photoelectron spectroscopy (XPS) was used to analyze the film surface. From the core loss peak positions only four elements were detected, the three metals (Fe, Tb, Dy) and O. Figure 2(b) compares the core-loss spectrum for as-prepared (dashed line) and O_2_ annealed (solid line) films for the energy window containing Fe and O, while Fig. 2(c) is for the energy window containing Tb and Dy. Quantitative analysis of the core-loss peak intensities established that the as-prepared films were metal oxides with a metallic composition of Me = Fe(66 at.%):Tb(10 at.%):Dy(24 at.%) and a metal to oxygen ratio of 2:3 with an inherent error of ~10% (<4% error on the individual elemental concentrations). The composition of the as-prepared film could therefore be expressed as Me_2_O_3-x_. The composition was found to be very homogeneous in its metal and oxygen concentration, with no evidence for any chemical segregation effects throughout the film. Similar analysis of the O_2_ annealed film gave an identical metal composition and a more fully oxygenated metal oxide Me_2_O_3_, consistent with a Fe to O ratio found in *Fe*_2_*O*_3_ EELS standards. From these measurements it was also clear that only the state of Fe changed upon O_2_ annealing while the Tb and Dy oxidation states did not change. XPS survey scans from the surface of the as-prepared and O_2_ annealed film (see supplemental material) yielded similar results in terms of the constituents present, i.e. the three metals and oxygen. A carbon peak was also detected and attributed to hydrocarbon contamination following exposure to atmosphere. Figure 2(d) shows the XPS spectra of the Fe 2p signal, which can be used to differentiate between metallic Fe and its various oxidized states. The as-prepared film (dashed line) showed the Fe to be predominantly in *Fe*^3+^ oxidation state, as evidenced by the known Fe^3+^ satellite peak in pure Fe_2_O_3_ in ref 13 near 718.8 eV (marked as *Fe*^3+^ on Fig. 2(d). Since the satellite position was shifted to slightly lower energies than in pure Fe_2_O_3_, some contribution from a lower oxidation state, such as Fe^2+^, was also evident. A small peak at 707.4 eV for the as-prepared film also indicated the presence of *Fe*^0^ [marked on Fig. 2(d)]. However, this unoxidized iron appeared to be discontinuously distributed on the film surface as TEM-EELS measurements did not detect any Fe^0^ in the film regions but only showed evidence for it within the PLD particulates. This was evidenced by the EELS spectra from the particulates, shown by the dotted line in Fig. 2(b), in which no oxygen O K peak was evident, implying that the iron was in metallic state in the particulates. Upon oxygen annealing, the intensity of the oxide peaks increased significantly while the metallic Fe peak disappeared, as seen in Fig. 2(d, dashed curve). The position of the satellite peak was closer to *Fe*^3+^ (as seen in Fe_2_O_3_) indicating that it was the primary oxidation state. The increase in oxygen concentration following annealing was also evidenced from XPS O 1s spectra [Fig. 2(e)] and corroborated the TEM results. In Fig. 2(f,g), the normalized XPS spectra corresponding to Tb 3d 5/2 and Dy 3d 5/2 levels, respectively, are shown for as-prepared (dashed line) and O_2_ annealed cases (solid line). The energy positions of these peaks were correlated very well with the signals from the respective oxides of the form Tb_2_O_3_ (which occurs at 1241.4 eV) and Dy_2_O_3_ (which occurs at 1298.9 eV)^14^ (and are indicated on the figure). Further, the XPS peak positions were unchanged between the as-prepared and annealing case suggesting that the oxidation state of the Lanthanide metals did not change upon annealing. In totality, these findings pointed to amorphous films in which the amount of O and Fe^3+^ increased in going from the as-prepared to the O_2_ annealed films, but without change in the oxidation state of the Lanthanide metals. The formation of an amorphous oxide film is not entirely surprising and we attribute it to the combination of forming an amorphous microstructure during the PLD process followed by its instantaneous oxidation upon exposure to air. Previous works focused on the magnetic behaviors of similar compounds (Fe-Tb-Dy) have shown that it is possible to synthesize amorphous metallic films by techniques such as sputtering^15^,^16^. Also, the surfaces of such compounds have been reported to oxidize fairly quickly in air leading to an oxide layer of thickness between 10 to 30 nm, while the bulk material continues to oxidize at a much slower rate, thus necessitating a capping layer to prevent degradation.

**Figure 3:**
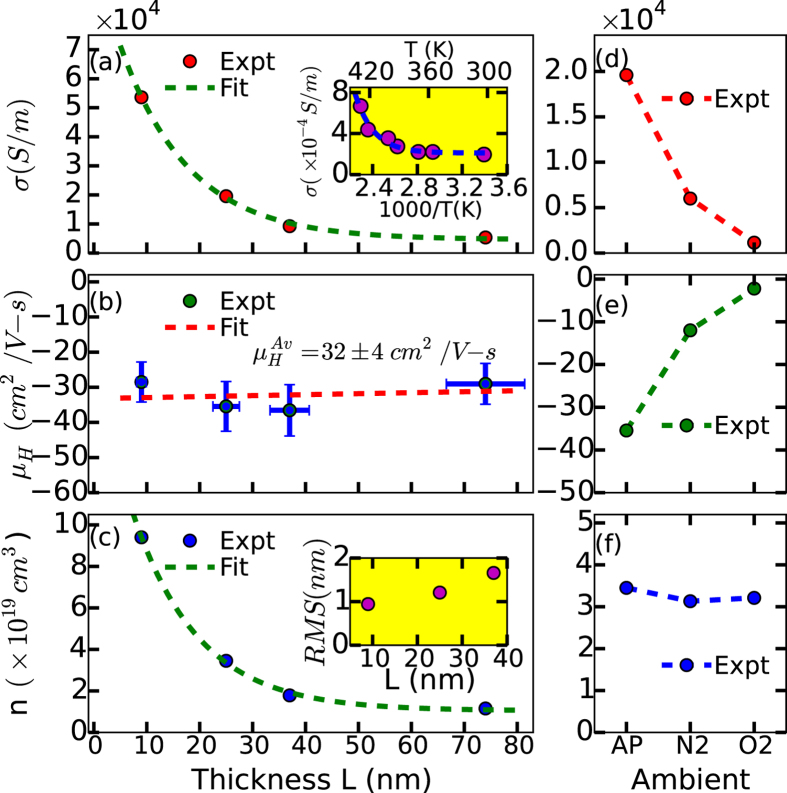
Transport properties of the as-prepared (a–c) and annealed films (d–f). (**a**) Dependence of conductivity on thickness of as-prepared films. Inset shows that the conductivity *σ* increased exponentially with temperature for a 25 nm as-prepared film. (**b**) The mobility of the as-prepared films showed n-type conductivity and its magnitude was relatively unchanged with thickness yielding an average value of 32 ± 4 cm^2^/V-s. (**c**) The electron carrier concentration in the as-prepared films decreased exponentially with increasing film thickness. This correlated with an increased surface roughness of the films (inset). (**d**) Conductivity change for 25 nm films following annealing in nitrogen (N_2_) or oxygen (O_2_) at 500 °C for 2 hrs. (**e**) Mobility change with annealing. (**f**) Carrier concentration remained relatively unchanged following annealing. In figures (**d**–**f**) the as-prepared films are indicated as AP while the O_2_ and N_2_ annealed films are marked as O2 and N2 respectively. The dashed lines in (**a**–**c**) correspond to best fits to the experimental data. The dashed lines in (**d**–**f**) correspond to guides to the eye.

Given the importance of transparent amorphous oxides to the electronics industry, we investigated the electrical properties of these films. Conductivity (*σ*) and Hall mobility (*μ*_*H*_) measurements were made using the 4-probe van der Pauw geometry for films between 9 and 74 nm in thickness deposited onto SiO_2_/Si substrates (i.e. Si containing a 400 nm thermally grown oxide layer). First we verified the nature of electrical conduction in these films by performing temperature-dependent conductivity measurements. As shown for a 25 nm as-prepared film in the inset of Fig. 3(a), the electronic transport confirmed a semiconductor-type material since the conductivity increased exponentially with temperature T. This result also ruled out any role of the discontinuously distributed metallic iron on the film surface in playing a role on the electronic properties. Figure 3(a) shows the room temperature conductivity for as-prepared films with various thicknesses. The measured conductivity ranged from ~5 × 10^3^ to 5 × 10^4^ S/m. These values are many orders of magnitude higher than that found in the constituent metal oxides noted in the previous section^12^,^17^. To understand the origin of the large conductivity (and its change with thickness), we measured the Hall mobility since it contributes directly to *σ* through the expression *σ* = *μ*_*H*_*ne*, where *n* is the carrier concentration, and *e* is the magnitude of electron charge. Figure 3(b) shows that the Hall mobility had a negative sign for all the as-prepared films studied, indicating an n-type semiconductor. Since its magnitude was relatively unchanged, it could not be responsible for the drop in conductivity with increasing thickness L. However, the measured value of 32 ± 4 cm^2^/V-s (averaged over the various as prepared films) was extraordinarily high, and comparable to the best known s-band amorphous oxide materials^2^. We were unable to detect any evidence for room temperature magnetism through hysteresis, coercivity or saturation behavior (see supplemental material), consistent with the fact that oxidation destroys magnetism in such alloys^16^. This also confirmed that the measured mobility was the *regular* hall mobility and therefore was directly contributing to the high electrical conductivity. The unchanging mobility with thickness implied that the change in conductivity with thickness was due to a change in the free carrier concentration, which was estimated from 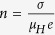 and is shown in Fig. 3(c). The values decreased from 9.4 × 10^19^ to 1.15 × 10^19^ cm^−3^ for the 9 to 74 nm films respectively. While such an effect has been reported before for ultrathin semiconducting films^18^, we speculate that the change in conduction could be partly attributed to the increase in surface roughness observed with increasing thickness of the films, as shown in the inset of Fig. 3(c).

The transport behavior following thermal treatments up to 500°C was measured for 25 nm thick films. Figure 3(d) shows that while the conductivity of the film decreased following the annealing, the magnitude of the drop was very different, ~3× decrease for the N_2_ case vs ~17× for the O_2_ case. To understand this change, the hall mobility was also measured, and, as shown in Fig. 3(b) n-type conductivity was observed in all cases, but the magnitude decreased from 35.4 cm^2^/V-s (for the as-prepared film) to 12 cm^2^/V-s for the N_2_ anneal and 2.2 cm^2^/V-s for the O_2_ case, which correlated very well with the magnitudes of the drop in conductivity shown in Fig. 3(d). Despite the changes following thermal annealing, the combination of conductivity and mobility observed in these as-prepared and thermally treated films was still orders of magnitude higher than in the component oxides (*Fe*_2_*O*_3_, *Tb*_2_*O*_3_, and *Dy*_2_*O*_3_) as we discuss next. These annealing results also hinted at a potential future path to control and modify the electronic and optical properties of this material.

## Discussion and Conclusion

Based on the substantial knowledge developed over the past decade, it is possible to summarize two common features found in all the ternary amorphous oxides that show high mobility (>10 cm^2^/V-s), such as the a-IGZO system. First, overlap of the large spherically symmetric *ns* levels involved in the metal cation bonding produces large s-conduction band curvature and consequently, a high mobility for carriers excited from the valence band formed by oxygen 2p states^7^,^9^. Second, the ternary oxide cannot have a mobility and/or conductivity far exceeding that of *all* of its constituent oxides, as exemplified by the relation between the ternary composition and the measured mobility values^2^,^10^. In fact, it can be stated that the primary reason to use a ternary system is to stabilize the amorphous microstructure.

Based on the dominant metal oxidation states measured by XPS investigations, it is tempting to interpret our observed optical and electronic behaviors in the above context, i.e. as arising from a mixture of the different semiconducting sesquioxides (i.e. *Fe*_2_*O*_3_, *Tb*_2_*O*_3_ and *Dy*_2_*O*_3_). But, all of these oxides are well-known insulators with no contribution from the s-band to their conductivity and mobility behaviors^19^. Semiconducting iron oxide (hematite or *α* − *Fe*_2_*O*_3_) is a charge transfer insulator in which the indirect optical band gap of 2.1 eV excites electrons from a valence band which is primarily comprised of the oxygen 2p levels into a conduction band which comes from the Fe 5d levels. In the band structure model, the extremely flat d-band (i.e. low curvature) results in very heavy electrons and the resulting low conductivity and mobility of <0.01 cm^2^/V-s^11^. Despite attempts to dope iron oxide, the best conductivity and mobility still remains at 4 S/m and <0.6 cm^2^/V-s respectively in high-quality crystalline thin films^17^, orders of magnitude lower than the values observed here for the as-prepared and thermally annealed amorphous films. The low mobility has also been explained as the consequence of conduction by polaron hopping, with polarons having a very large effective mass due to the strong interaction between electrons and the lattice in such ionic crystals^20^. The Lanthanide oxides are materials with potential applications as high-K dielectrics because of their electrically insulating nature and large band gaps^12^,^21^. These oxides also have valence bands showing primarily O 2p character and conduction bands coming from the 5d levels. However, their unique feature is that the 4f levels can introduce filled and/or empty states at different positions with respect to the optical band-gap^22^. Nevertheless, the extremely flat nature of the f-levels as well as the d-conduction band again results in exceedingly low room temperature conductivity (<10^−12^ *S*/*m*) and electron mobility values (<2 cm^2^/V-s)^12^.

Clearly, the s-subshells of these metal cations (Fe, Tb and Dy) are highly unlikely to contribute to the conduction band^19^. Therefore, this ternary amorphous oxide has a profoundly different origin of its high mobility as compared to the existing s-band high mobility amorphous oxides. This material also shows an emergent behavior because its conductivity and mobility far exceeds that of its constituent oxides. Our future work towards identifying the origin of this condensed matter behavior will focus on the hypothesis that there is a strong interaction between the Fe 5d and Lanthanide 4f levels. We conclude by speculating that the electronic properties of this material could be indirect evidence for an interesting new band structure physics arising from the interaction of the transition and lanthanide metal cations. Additionally, this material could also be technologically relevant because it shows a combination of electrical conductivity and mobility that rivals that of the best known Indium-based transparent semiconducting oxides. Perhaps the most important implication of this work is that, given the vastly greater number of transition metals which have unfilled d-levels as compared to metals which show (n-l)d^10^ns^0^ behavior, it presents the intriguing possibility of creating a whole new class of functional oxide electronic materials.

## Methods and Techniques

### A. Material synthesis and processing

Thin films of Fe:Tb:Dy were deposited using the pulsed laser deposition (PLD) technique. Terfenol-D, which has a composition of (Tb_0.3_Dy_0.7_)Fe_1.92_, was used as a PLD target to deposit the thin films. This material was purchased from Etrema Products Inc., USA. The films were deposited on quartz or SiO_2_/Si wafers having 400 nm of thermally grown oxide. Before deposition, the substrates were cleaned by sonicating them in acetone, isopropanol and DI water for 30 min each and then dried with nitrogen and stored. PLD was done using a Spectra Physics injection seeded Lab-130–50 Nd:YAG laser with wavelength of 266 nm, a pulse width of 9 ns and repetition rate of 50 Hz in a ultra high vacuum at a base pressure of 5 × 10^−8^ Torr. A laser energy density of 0.56 J/cm^2^ was used for deposition. Following the deposition the samples were removed and exposed to ambient air and stored in metallic sample boxes under ambient conditions. The annealing of the samples were done either in oxygen rich (air) or nitrogen rich environment (99.9% purity of N_2_ gas supplied by Airgas Inc., Knoxville, USA) in a programmable oven from MTI corporation (model no.: OTF-1200X) at 500 °C for 2 hours. The contact pads for electrical measurements were made by masking the samples with Al foil and then depositing Ag pads on the amorphous films. The Ag pads were deposited using the e-beam evaporator at a base pressure of 2 × 10^−8^ Torr and were approximately 40 nm thick.

### B. Surface characterization

Scanning electron microscopy (SEM) was used to obtain the morphology of the surface of the films deposited using PLD. The imaging was done using a Zeiss Merlin SEM operated at 2 kV using an inlens detector. Roughness measurements of the as-prepared films were made by atomic force microscopy (AFM). Areas of 4 × 4 *μm*^2^ were scanned for the various films and root mean square (rms) roughness was calculated by averaging over multiple (up to 256) line profiles at different areas. The film roughness measurements were done using Nanonics Multiview 1000 AFM, which was operated in line-by-line tapping mode at a resolution of 256-by-256 and a rate of 8 ms per point. The cantilever tip had a radius of curvature measuring less than 40 nm.

### C. Material characterization

#### TEM

The TEM sample for as-prepared film investigation was made by depositing 9 nm thick films onto ultrathin C substrates on mica. The film/C system was floated off from mica by immersion in water, yielding the electron transparent material (as described by Sachan *et al*. in ref. 23). The O_2_ annealed sample was prepared by depositing a 25 nm thick film onto electron transparent Si_3_N_4_ grids (which had thickness 10 nm) with window size of 100 × 100 *μm*^2^ supplied by Norcada, Canada. Oxidation of this sample was performed as described earlier. High resolution TEM images and diffraction patterns were taken in a Zeiss Libra 200 MC at an acceleration voltage of 200 kV, while the Z-contrast images and EELS spectra were taken with an aberration corrected (Nion, Inc.) dedicated STEM VG 501 UX operated at 100 kV. This instrument is equipped with a cold field emission electron source and a Gatan Enfina EELS spectrometer.

#### XPS

XPS measurements were carried out at room temperature by using a SPECS Focus 500 monochromated Al K*α* X-ray source operated at 380 W and a SPECS PHOIBOS-150 hemispherical electron analyzer at normal emission and 40 eV pass energy. Relative atomic concentrations were taken from comparison of Dy 3d, Tb 3d_5/2_, O 1s, and Fe 2p_3/2_ core levels, analyzed and corrected for sensitivity and transmission factors in CasaXPS software.

#### XRD

As-deposited and annealed thin films were characterized using grazing incidence X-ray diffraction (GIXRD). These measurements were performed using a Panalytical X’ Pert3 MRD X-ray diffractometer equipped with Cu K*α* source (1.54059 Å) radiation and a Xe-proportional detector. The GIXRD patterns were recorded in a 2*θ* scanning mode using a parallel beam mirror on the incident beam side and a parallel plate collimator of 0.27 divergence on the diffracted beam side. A combination of beam mask and divergence slits was selected to illuminate the sample surface without illuminating the sample holder. In order to avoid diffraction from the sample holder the samples were mounted on a 2-inch single crystal silicon wafer oriented slightly off axis. The GIXRD patterns were collected in the 2*θ* range between 10–90° with a step size of 0.02° and step time of 7 sec/step.

### D. Optical properties

The optical properties of the ternary amorphous oxide semiconductor were measured using HR2000 + ES spectrometer from Ocean Optics in transmission mode. The Tauc plots were generated by first converting transmission values (T in %) to absorption spectra using Beer-Lambert’s law and then dividing by the film thickness (L) to convert to absorption coefficient 
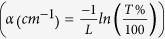
 as a function of wavelength of light. The optical band gaps were then calculated by plotting Tauc plots with y-axis as (*αhv*)^1/*m*^ as a function of *hv* (the photon energy given by product of Planck’s constant *h* and frequency *v*). Tangents were drawn at the interband absorption region (rapid rise in spectrum) and were extrapolated to cut the x-axis, which gave the optical band gap values. *m* = 2 was used to obtain direct band gap values.

### E. Magnetic properties

The magnetic properties of the as-prepared and annealed films were studied using surface magneto-optical Kerr effect (SMOKE) technique. The SMOKE measurements were done in the longitudinal orientation using an s-polarized laser beam of 633 nm wavelength making 12.6° angle of incidence with the normal to the substrate plane.

### F. Electrical properties

For measuring the electrical properties, silver pads were deposited on the four corners of the sample using e-beam evaporation, as described previously. Gold wires were then attached to the silver pads using silver epoxy paste. A Keithley 2400 sourcemeter was used to measure the sheet resistance and the hall mobility of the deposited amorphous oxide. Sheet resistance was measured using the van der Pauw method, where probe contacts are made at the four corners of the sample. Current was supplied at two adjacent contact points while voltage was measured at the two remaining contact points, i.e. if the four contacts were numbered 1, 2, 3, and 4, current was supplied between 1 and 2 (*I*_12_), while voltage was measured between 4 and 3 (*V*_43_) to get resistance *R*_12,43_. In this way, the current direction was changed to cover all four sides, making sure to reverse the current direction on each side, resulting in eight total measurements. The four-probe resistance was measured by the Keithley sourcemeter working in 4-wire sensing mode. A LabVIEW code was written to collect data from the Keithley for 1 minute and then display the average value. This method of data collection ensured noise-compensated resistance values. After all the eight resistance values were measured (*R*_12,43_; *R*_21,34_; *R*_34,21_; *R*_43,12_; *R*_41,32_; *R*_14,23_; *R*_23,14_; *R*_32,41_), the following formula was used to calculate the sheet resistance: exp(−*πR*_*A*_/*R*_*S*_) + exp(−*πR*_*B*_/*R*_*S*_) = 1, where *R*_*A*_ = (*R*_12,43_ + *R*_21,34_ + *R*_34,21_ + *R*_43,12_)/4, *R*_*B*_ = (*R*_41,32_ + *R*_14,23_ + *R*_23,14_ + *R*_32,41_)/4, and *R*_*S*_ is the sheet resistance. The resistivity was calculated as the product of the sheet resistance and the film thickness.

Hall measurements were made by supplying current along the contacts 3 and 1, and measuring the voltage between 4 and 2. For each value of current, the magnetic field was varied and the corresponding Hall voltages were measured. Just like for sheet resistance measurements, a LabVIEW program was used to collect data to compensate for noise and drift. A plot was then made of Hall voltage vs applied magnetic field and a straight line fit was applied to it to obtain the slope of the plot. The hall mobility, *μ*, was then calculated using: 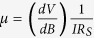, where I is the current supplied and *R*_*S*_ is the sheet resistance. The process was repeated with at least three different current values to obtain reliable Hall mobilities. The carrier concentration, n, was calculated as 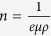, where e is the charge on an electron, and *ρ* is the resistivity.

Sheet resistance was also measured as a function of temperature using Keithley 2400 sourcemeter. Although the same 4-wire sensing mode was used as for sheet resistance measurement, the contacts for current supply and voltage measurement were fixed to one configuration so as to not disturb the system while the film was being heated with an IR lamp. The temperature was measured periodically using a laser temperature sensor and the corresponding resistance value was noted from the sourcemeter.

## Additional Information

**How to cite this article**: Malasi, A. *et al*. Novel Iron-based ternary amorphous oxide semiconductor with very high transparency, electronic conductivity, and mobility. *Sci. Rep*. **5**, 18157; doi: 10.1038/srep18157 (2015).

## Supplementary Material

Supplementary Information
