# Supplementary material for: Novel Iron-based ternary amorphous oxide semiconductor with very high transparency, electronic conductivity, and mobility
Source: Sci Rep. 2015 Dec 16;5:18157. doi: 10.1038/srep18157 (PMC4680984; doi:10.1038/srep18157)
Supplement: Supplementary Information [file srep18157-s1.pdf]

Supplemental Information for “An iron oxide based transparent amorphous semiconductor with extraordinary electronic conductivity and mobility”

A. Malasi,[1] H.Taz,[2] A. Farah,[3] M. Patel,[2] B. Lawrie,[4] R. Pooser,[4] A. Baddorf,[5,6] G. Duscher,[3,6] and R. Kalyanaraman[1,2,3*]

[1]Department of Chemical and Biomolecular Engineering, University of Tennessee, Knoxville, Tennessee, 37996, USA
[2]Bredesen Center, University of Tennessee, Knoxville, Tennessee 37996, USA
[3]Department of Material Science and Engineering, University of Tennessee, Knoxville, Tennessee 37996, USA
[4]Quantum Information Science Group, Computational Sciences and Engineering Divison, Oak Ridge National Laboratory, Oak Ridge, TN 37831, USA
[5]Center for Nanophase Materials Sciences, Oak Ridge National Laboratory, Oak Ridge, TN 37831, USA
[6]Material Science and Technology Division, Oak Ridge National Laboratory, Oak Ridge, TN 37831, USA

Supplemental Information

A. Film roughness

Typical AFM line scans from the as-prepared films of 9, 25 and 37 nm thickness are shown in Fig. 1. From 256 such lines scans for each film thickness, the average root mean square (RMS) roughness was estimated as 0.944, 1.208 and 1.659 nm for 9, 25 and 37 nm films, respectively.

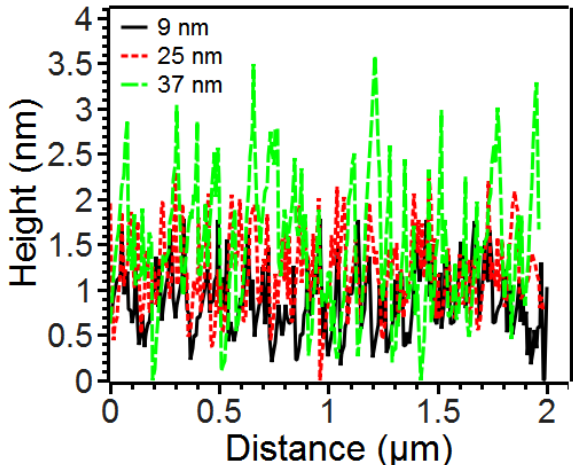

Figure 1: Surface roughness profile of as-deposited films measured using AFM.

*ramki@utk.edu

B. GIXRD measurements of films

Fig. 2(a) shows the GIXRD scan of the Terfenol-D target which comfirmed that the starting materials had the right metallic composition of terfenol-D. In Fig. 2(b) GIXRD scans of 25 nm thick films in as-prepared and the two annealed cases is shown and compared to GIXRD from the SiO_2/Si substrate. Since no features were evident from the films we concluded that they had an amorphous microstructure. The indexing of the XRD peaks was done using the ICSD database [1]. For the indexing of Terfenol-D, indexing till 70° was done with the help of ICSD database, while the remainder was based on published literature [2, 1].

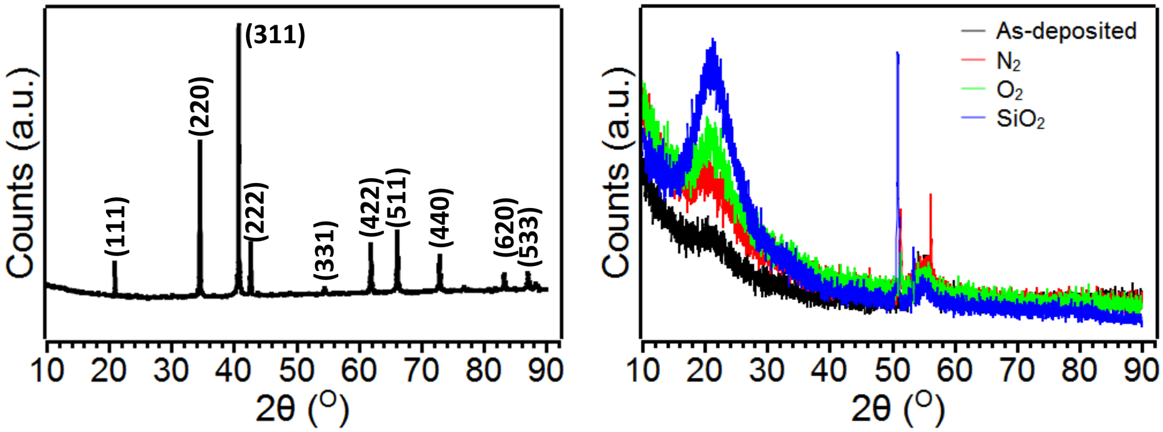

(a) (b)

Figure 2: *(a) Shows the XRD of the Terfenol-D target. Indexing of the various peaks showed that the targert was consistent with hte original terfenol-D composition. (b) Comparison of GIXRD scans of as-prepared, nitrogen annealed, and oxygen annealed films of 25 nm thickness as well as the underlying substrate which was SiO_2(400 nm)/Si. The only features evident from the films were the underlying substrate features, as indicated on the figure. This confirmed that the films were amorphous.*

E. Tauc plot measurement

Fig. 3(a), the Tauc plot of the three as-prepared films (with thickness of 9 , 25 and 37 nm) is shown. The Band gap value was 3, 2.82, and 2.7 eV respectively. From these measurements, the average band gap value was obtained as 2.85±0.14 nm.

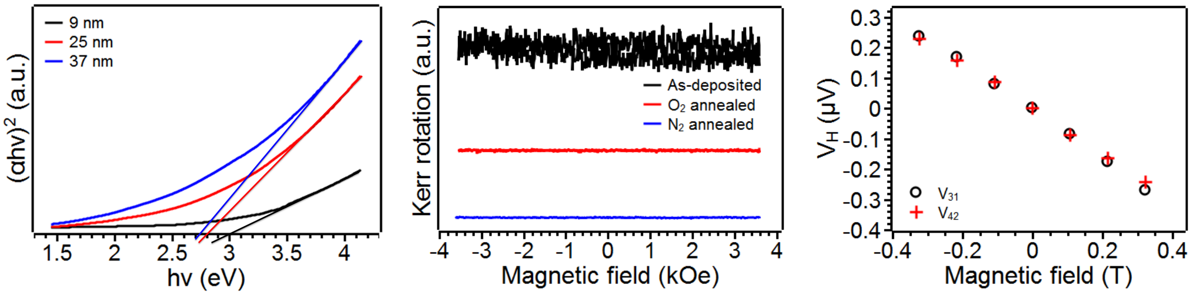

(a) (b) (c)

Figure 3: (a) Tauc plot of as-deposited films. (b) Kerr rotation measurements of as-prepared and annealed films. (c) Plot of Hall voltage as a function of applied magnetic field for a 74 nm thick as-prepared film studied for two different voltage configurations for an applied current of 100 μA.

F. Magnetic measurements by SMOKE

The as-prepared and annealed films showed no Kerr rotation within the detection limit of the SMOKE system. The Kerr plots for the as-prepared and O_2 annealed film with 25 nm thickness are shown in Fig. 3(b). Similar results were obtained for all other films (i.e. as-prepared 9, 37, 74 nm) and N_2 annealed sample. From this it was concluded that no room temperature magnetism was present in these films.

G. Hall Effect Measurement

The hall voltage versus magnetic field data was used to calculate the hall coefficient and mobility. The hall voltage versus magnetic field curve was linear, and a typical case is shown in Fig. 3(c) for the 74 nm thick as-prepared film. Similar linear curves were obtained for all other samples measured (as prepared and annealed).

C. XPS Measurements

Survey scans from the surface of as prepared and O_2 annealed films are shown in Fig. 4. Analysis based on the energy position of the various features compared to standards noted in the Handbook for XPS [3] revealed that Fe, Tb, Dy and O were the primary components while hydrocarbon peals were also present due to handling conditions.

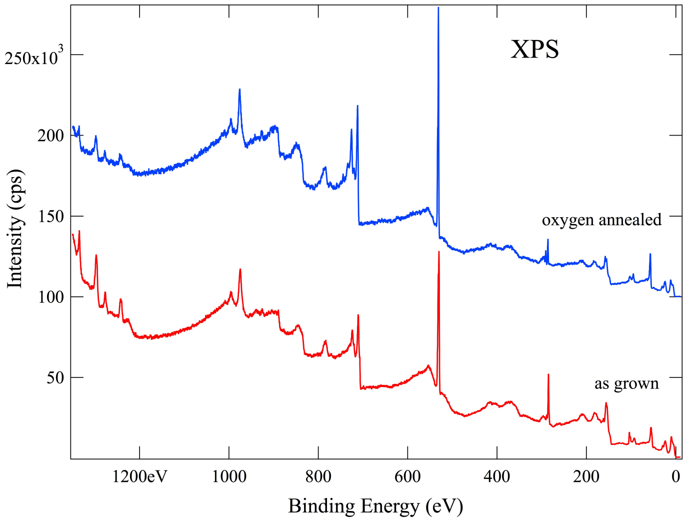

Figure 4: XPS survey scans to determine elements present in the as-prepared and annealed films.

D. SEM Measurement

In the as-prepared films large surface nanoparticles or PLD chunks could be seen on the surface of the films, and this was characteristic of the PLD process (as shown in manuscript Fig. 1c). Upon annealing in air there was a decrease in the size of particulates presumably due to oxidation, as evident from the SEM images of the annealed samples shown in Fig. 5(a).

E. TEM and EELS measurements

Fig. 5(b) shows the micrograph of a 25 nm thick film made by deposition onto a Si_3N_4electron transparent grid and annealed in O_2 at 500°C for 2 hours. Like the as-prepared films, the microstructure was amorphous for this film as well.

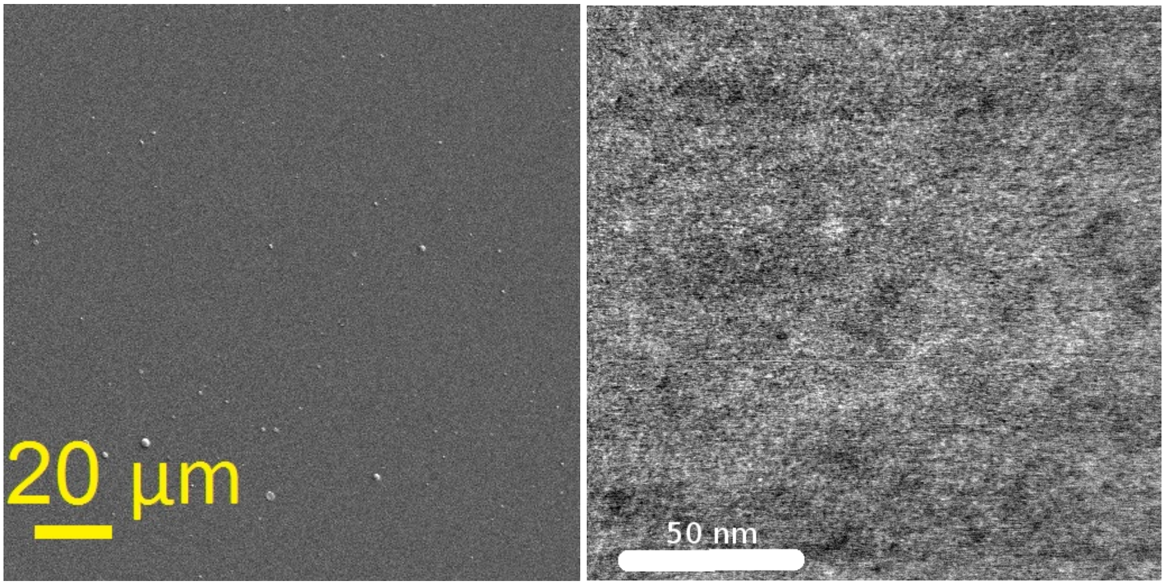

(a) (b)

Figure 5: *(a) SEM micrograph of the surface of an O_2 annealed film shows a smooth morphology and a decrease in the nanoparticles from the PLD process in comparison to the as-prepared films (shown in manuscript Fig. 1(c). (b)* TEM micrograph of O_2 annealed film.

References

[1] “Icsd database.”

[2] G. Ausanio, A. C. Barone, V. Iannotti, P. Scardi, M. Dincau, S. Amoruso, M. Vitiello, and L. Lanotte, “Morphology, structure and magnetic properties of (tb0.3dy0.7fe2)100-xfex nanogranular films produced by ultrashort pulsed laser deposition,” *Nanotechnology*, vol. 17, pp. 536–542, 2006.

[3] J. F. Moulder, W. F. Stickle, P. E. Sobol, and K. D. Bomben, *Handbook of X-Ray Photoelectron Spectroscopy*. Physical Electronics, Inc, Eden Prairie, MN, USA, 1995.
